# Characterization of *Qu*-aroma of medium–high temperature *Daqu* from different production areas using sensory evaluation, E-nose, and GC–MS/O analysis

**DOI:** 10.1186/s40643-025-00863-y

**Published:** 2025-04-26

**Authors:** Jie Deng, Jia Zheng, Xing Li, Dan Huang, Guangbin Ye, Huibo Luo

**Affiliations:** 1 Key Laboratory of Brewing Molecular Engineering of China Light Industry, Beijing Technology and Business University, Beijing, 100048 China; 2 Liquor Making Biotechnology and Application Key Laboratory of Sichuan Province, Sichuan University of Science and Engineering, Yibin, 644000 People’s Republic of China; 3 Wuliangye Yibin Co.Ltd, Yibin, 644007 People’s Republic of China

**Keywords:** Medium–high temperature *Daqu*, *Qu*-aroma, Volatile compounds, Sensory analysis

## Abstract

*Qu*-aroma is a critical quality indicator for medium–high temperature Daqu (MT-*Daqu*). Due to the differences in regional and environmental conditions, these differences are likely to cause variations in the *Qu*-aroma of MT-*Daqu*. Despite this, comprehensive research on the variations in *Qu*-aroma across different regions has been lacking. This study aims to address this gap to analyze the differences in *Qu*-aroma characteristics of MT-*Daqu* from seven distinct production areas. Sensory analysis identified seven broad aroma categories and 25 specific aromas characterizing the *Qu*-aroma of MT-*Daqu* samples. Chen aroma and roasted aroma were notably more prevalent in MT-*Daqu* samples from Sichuan compared to those from other regions (*P* < *0.05*). In total, 123 volatile compounds were detected in MT-*Daqu*, with 42 classified as aroma-active compounds. Among these, 21 aroma-active compounds were identified as markers distinguishing Sichuan MT-*Daqu* from those produced in non-Sichuan regions, showing significant differences in their concentrations (*P* < *0.05*). Additionally, six major aroma-active compounds were found to significantly contribute to the aroma differences between Sichuan and non-Sichuan MT-*Daqu*. This study delineates the distinct *Qu*-aroma characteristics of MT-*Daqu* from various SAB production areas. The insights gained from this research offer valuable guidance for optimizing flavor profiles in MT-*Daqu* production.

**Graphical Abstract:**

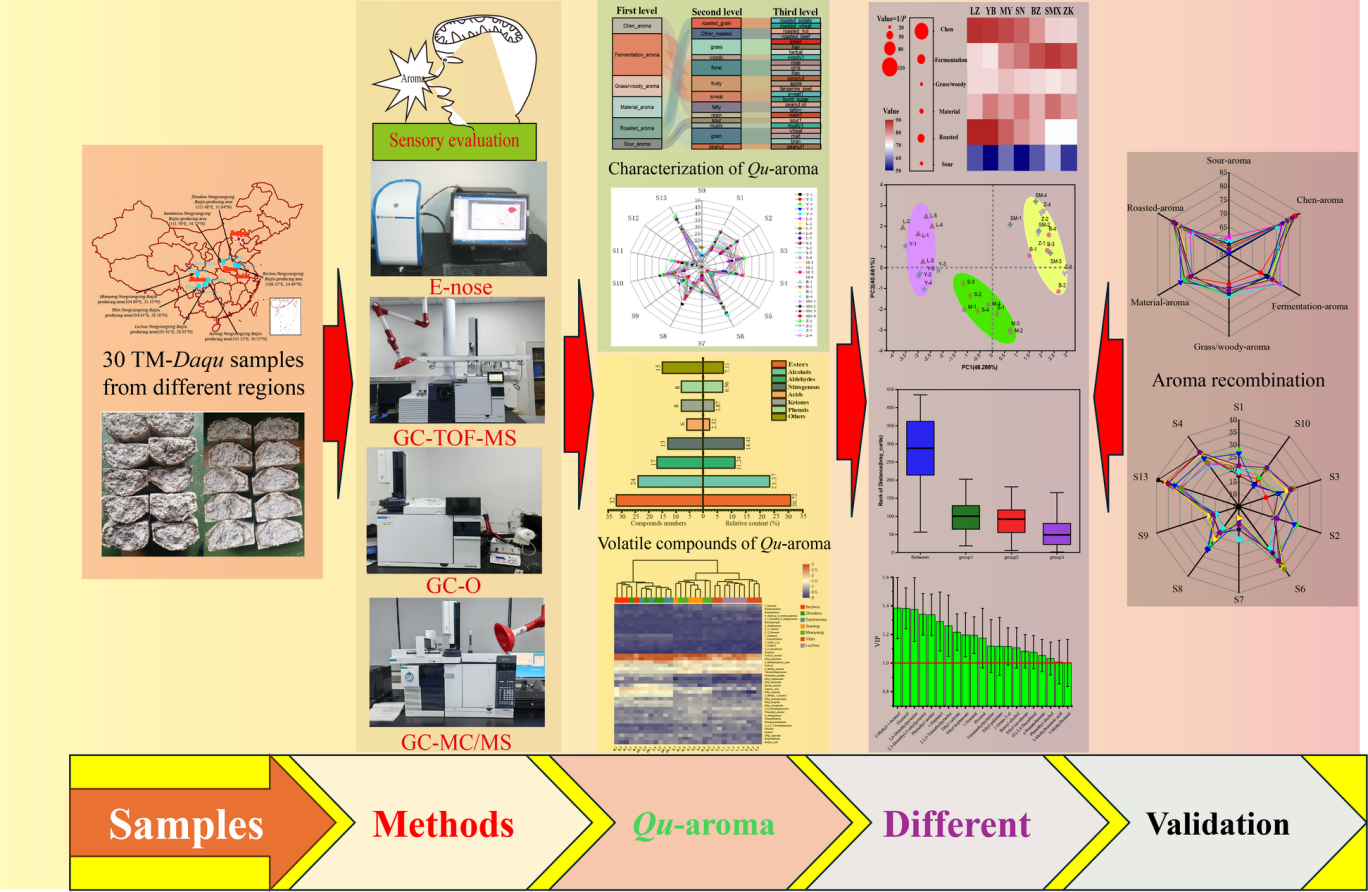

**Supplementary Information:**

The online version contains supplementary material available at 10.1186/s40643-025-00863-y.

## Introduction

Chinese *Baijiu*, one of the six major distilled spirits globally, is renowned for its distinctive flavor profile (Liu & Sun 2018). The phrase “*Qu* is the bone of *Baijiu*” highlights the critical role of *Qu* in the *Baijiu* brewing process. High-quality *Baijiu* production relies heavily on *Daqu* (Deng et al. 2023), a key fermentation starter. *Daqu* is made from wheat that is first crushed, mixed with water, pressed into brick blocks, and then cultured and fermented under controlled temperature and humidity conditions (Liu et al. 2023a, b, c). Following fermentation, *Daqu* is air-dried and stored as brick-shaped blocks (Liu and Sun 2018). Based on peak fermentation temperatures, *Daqu* is categorized into four types: high-temperature *Daqu*, medium–high-temperature *Daqu* (MT-*Daqu*), medium-temperature *Daqu*, and low-temperature *Daqu*. Currently, MT-*Daqu* is widely used as a fermentation starter in the production of *Strong Aroma-type Baijiu* (*SAB*). In 2023, *SAB* production saw revenues exceeding $40 billion, with over 2.5 million tons of MT-*Daqu* utilized. Sichuan is the largest *SAB*-producing region, accounting for over 60% of both production and sales, followed by Jiangsu, Anhui, Henan, and other regions.

*Daqu* contains a diverse array of microorganisms, enzymes, and compounds that are crucial for the saccharification and fermentation of grains, thus earning its designation as a traditional *Baijiu* fermentation starter (Niu et al. 2018). The manufacture of *Daqu* involves microbial enrichment, culture, and screening, with the environmental conditions during production playing a direct role in shaping the microbial community and the overall quality of *Daqu*. Key environmental factors influencing the fermentation of MT-*Daqu* include temperature, acidity, oxygen, carbon dioxide, humidity, moisture, and other conditions. Temperature and acidity are the primary factors affecting bacterial community succession, while temperature and humidity are crucial for fungal community succession during MT-*Daqu* fermentation (Ma et al. 2022a, b; Zhu et al. 2022). Additionally, studies have demonstrated that biological heat is a significant environmental driver influencing the microbial community in MT-*Daqu*. The peak temperature is a critical control parameter and one of the most important microbial indicators in the MT-*Daqu* fermentation process (Xiao et al. 2017).

Moreover, physicochemical properties, enzyme activity, microorganisms, and *Qu*-aroma are critical index for evaluating the quality of MT-*Daqu*. The “General Methods of Analysis for *Daqu*” (QB/T 4257-2011) includes four key evaluation indexes for *Daqu*. Multi-omics studies have shown that MT-*Daqu* is rich in enzymes, making it a major source of enzyme resources, especially those that function under extreme conditions. These enzymes are crucial for the saccharification and fermentation processes of grains (He et al. 2023; Yi et al. 2018). Previous research has provided an in-depth analysis of the production process, physicochemical properties, enzymes, and microorganisms involved in MT-*Daqu*, laying a theoretical foundation for optimizing preparation conditions and control parameters (Deng et al. 2023; Kang et al. 2022; S. Ma et al. 2022a, b; Mu et al. 2023). Additionally, the current production process of MT-*Daqu* is well described (Figure S1A). Meanwhile, The importance of *Daqu* in *Baijiu* brewing is well-established, with its role as a saccharifying agent and fermentation starter being critical to the production process (Liu et al. 2023a, b, c; Yang et al. 2024a, b). Recently, leading *Baijiu* producers have increasingly focused on the impact of *Qu*-aroma on the final aroma profile of *Baijiu*. The Chinese standard GB/T 10781.4–2024 has formally recognized *Qu*-aroma as a key metric for evaluating *Baijiu* aroma, attributing *Qu*-aroma to the fermentation process involving *Daqu* (Chen et al. 2024). However, while considerable attention has been given to the process, microorganisms, and enzymes in MT-*Daqu*, there remains a gap in research concerning its substance composition, particularly the composition of *Qu*-aroma in MT-*Daqu*.

In the production of SAB, MT-*Daqu*, grains, and fermented grains are mixed and fermented together before distillation to produce the base *Baijiu*. In the Baijiu flavor wheel, *Qu*-aroma is classified as the raw material aroma. As a result, *Qu*-aroma is imparted to the final product. A study employing headspace solid-phase microextraction (HS-SPME) combined with gas chromatography-mass spectrometry (GC–MS) identified 139 volatile compounds in the flavor profile of MT-*Daqu*. These include 36 esters, 22 alcohols, 14 aldehydes, 12 nitrogenous compounds, 9 terpenes, 8 phenols and furans, 7 acids and ketones, 4 lactones, 3 sulfur compounds, and 9 others (Wang et al. 2022). Further research on MT-*Daqu* aroma has identified four primary aroma categories: fermentation aroma, material aroma, aging aroma, and others. These encompass 10 secondary aromas and 52 tertiary aromas, with 37 aroma-active volatile compounds identified using thin-film (TF)-SPME-GC-O-MS (Yang et al. 2024a, b). These studies have provided a detailed characterization of the composition and aroma characteristics of *Qu*-aroma in MT-*Daqu*. Despite these advances, geographical and environmental differences in MT-*Daqu* production have been noted, leading to variations in the structure and potential functions of microbial communities and volatile compounds in MT-*Daqu* samples from different regions (Ma et al. 2022a, b). This suggests that *Qu*-aroma in MT-*Daqu* may differ across regions, yet there remains a lack of comprehensive research on regional variations in the *Qu*-aroma of MT-*Daqu*.

In the present study, MT-*Daqu* samples were collected from four *SAB*-producing areas in Sichuan and three *SAB*-producing areas outside Sichuan. *Qu*-aroma in MT-*Daqu* was analyzed using both artificial and electronic noses, alongside GC–MS for the qualitative and quantitative analysis of volatile components. Additionally, GC-O was utilized to identify aroma-active compounds contributing to *Qu*-aroma across different regions. Recombination experiments were also conducted to evaluate regional differences in *Qu*-aroma among MT-*Daqu* samples. This study provides a comprehensive understanding *Qu*-aroma characteristics and composition of volatile compounds of MT-*Daqu* from various SAB production areas. This aims to provide scientific evidence for the development of flavor-oriented production control technologies and lay a foundation for understanding the influence of *Qu*-aroma on SAB fragrance.

## Materials and methods

### Sample collection

Samples were collected from seven SAB-producing areas: Yibin, Luzhou, Suining, Mianyang, Bozhou, Sanmenxia and Zhoukou. Five parallel samples were collected from Yibin and Luzhou production areas, labeled Y1-5 and L1-5, respectively. Four parallel samples were collected from Suining, Mianyang, Bozhou, Sanmenxia and Zhoukou production areas, labeled S1-4, M1-4, B1-4, SM1-4, and Z1-4, respectively. The detailed location information was provided in Figure S1B. The samples of MT-*Daqu* were collected using the five-point method, these samples were accepted as qualified *Daqu*, each sample weighed at least 5 kg. Of this, 200 g were stored in liquid nitrogen for volatile compound analysis, while the remaining samples were used for E-nose analysis and sensory evaluation.

### Chemicals

Chemicals used in this study were categorized into four groups based on their properties and semi-quantitative content. Group A: ethyl butyrate, ethyl heptanoate, ethyl isovalerate, ethyl valerate, ethyl caproate, ethyl phenylacetate, ethyl palmitate, phenethyl acetate, 3-methyl-1-butanol, butyric acid, 3-methylbutanoic acid, caproic acid, furfuryl alcohol, phenethyl alcohol, benzyl alcohol. These were purchased from ANPEL Laboratory Technologies Co., Ltd. (Shanghai, China) with a purity greater than 99%. Group B: 1-heptanol, 2-ethylhexanol, ethyl benzoate, 1-octanol, 1-octen-3-ol, phenylethylene, and I-caryophyllene. These were obtained from ALADDIN Chemical Reagent Co., Ltd. (Shanghai, China) with a purity greater than 98%. Group C: phenylethylene, γ-nonanolactone, 4-ethylguaiacol, butyraldehyde, hexanal and 1-nonanal (≥ 98% purity), 3-methylbutanal, 2,6-dimethylpyrazine (≥ 97% purity), tetramethylpyrazine (≥ 97% purity), phenylacetaldehyde, γ-butyrolactone, and furfural (≥ 95% purity). These were purchased from Sichuan Victory Biological Technology Co., Ltd. (Chengdu, China). Group D: guaiacol, (*E*)-2-nonenal, (*E*)-2-octenal, 2,3-dimethyl-5-ethylpyrazine, 4-hydroxy-4-methoxystyrene, 2,3,5-trimethylpyrazine, benzaldehyde (≥ 98% purity), and (e)-2,4-decadienal (≥ 95% purity). These were also obtained from Sichuan Victory Biological Technology Co., Ltd. (Chengdu, China). Additionally, n-alkanes (C7-C30), the internal standards n-butyl acetate, and 2-acetylpyridine were sourced from Tan-Mo Technologies Co., Ltd. (Beijing, China). Liquid nitrogen and nitrogen gas (99.999% purity) were provided by Chengdu Xinyuan Chemical Co., Ltd. (Chengdu, China).

### Sensory evaluation

Sensory evaluation was performed following the Chinese standard GB/T 16291.1–2012 and the industry-specific MT-*Daqu* evaluation method. Twelve evaluators, all experienced in *Daqu* flavor analysis, were recruited from Sichuan University of Science and Engineering (Yibin, Sichuan, China). The group comprised 7 males and 5 females, with an average age of 34 years, each having over 5 years of experience in sensory evaluation of *Daqu*. The evaluators were assessed for their odor perception, sample discrimination ability, consistency, and repeatability in a standard food odor evaluation laboratory, using methods described in previous studies (Duan et al. 2024). As the evaluators were already familiar with *Daqu* aroma, no additional training in sensory analysis or aroma identification was required. However, they were introduced to the establishment and use of evaluation scales.

For the sensory evaluation, MT-*Daqu* samples were portioned into 20 g servings, with at least 10 servings per sample. Each sample was assigned a 3-digit random identifier. During each evaluation session, samples were randomly selected based on these identifiers. The evaluators initially conducted a blind assessment of the 7 MT-*Daqu* samples, describing both the category and intensity of the aroma, following previously established methods (Yu et al. 2023). Data on the MT-*Daqu* aroma were then compiled and organized by categorizing the aroma compounds into hierarchical levels. Sensory descriptors were selected and developed using methods outlined in previous studies (Yu et al. 2023), and evaluators re-evaluated the samples to confirm the completeness of flavor identification and the accuracy of classification. Finally, based on the primary aroma categories identified, the evaluators rated the aroma intensity of 30 samples using a 100-point scale. The sensory evaluation was conducted blindly, with evaluators unaware of specific sample details. Each sample was evaluated in triplicate, and the results were averaged for statistical analysis.

### Electronic nose analysis

An iNose E-nose (iSenso, USA) was utilized to evaluate the *Qu*-aroma characteristics of MT-*Daqu* samples. In brief, 20 g of each sample was placed in a 150-mL glass vial with a rubber stopper, then incubated in a 50 °C water bath for 30 min to equilibrate. Following the manufacturer’s instructions, the device was calibrated with zero gas, and the sensors were reset three times. Gas samples were drawn from the headspace of the vials at a flow rate of 200 mL/min, with a sampling time of 6 s and a detection time of 80 s. The E-nose system featured 20 sensors; for this study, 14 sensors were selected, as detailed in Table S1. The maximum value and maximum slope of the sensor responses were analyzed. A radar plot was created to visualize flavor intensity, and principal component analysis (PCA) was employed to assess differences among the samples.

### Extraction of volatile compounds

#### Solid-phase microextraction (SPME)

Volatile compounds from MT-*Daqu* samples were extracted following a method (Wang et al. 2022) with minor modifications. Briefly, 5 g of the sample was placed in a 40-mL centrifuge tube with 20 mL of ultrapure water and subjected to ultrasonic treatment for 1 h. The mixture was then soaked for 12 h before centrifugation at 7,000 rpm for 5 min. The supernatant was discarded, and the loss of extract was calculated by weighing the remaining supernatant and comparing it to the initial weight of ultrapure water added. The data were used to convert the volatile compounds. Next, 5 mL of the supernatant and a stir bar were transferred to a 15-mL glass vial, and 1 μL of 2-acetylpyridine (2.12 μg/μL) was added as an internal standard. The vial was sealed and placed in a 50 °C water bath, stirring at 180 rpm for 30 min. SPME fibers (DVB/CAR/PDMS, 50/30 μm, Supelco, Bellefonte, PA, USA) were then inserted into the headspace vial to adsorb volatile compounds for 30 min. After adsorption, the fibers were inserted into the GC inlet for 5 min to release the adsorbed volatile compounds, which were subsequently analyzed qualitatively and semi-quantitatively.

#### Liquid–liquid extraction (LLE)

Volatile compounds from MT-*Daqu* were extracted as described in Sect. "Solid-phase microextraction (SPME)", with the extract volume being recorded. The LLE method used, with minor modifications, was adapted from a published paper (Wang et al. 2020; Zhao et al. 2018). In this method, n-hexane was added at a ratio of 1:1.5 to the extract volume. The organic phase was collected after two rounds of extraction. This organic phase was then dried over anhydrous Na_2_SO_4_ and concentrated to 1000 μL using a low-temperature vacuum concentrator (RayKol, Xiamen, China). The concentrated extract was immediately analyzed using GC–MS/MS for precise quantification of volatile flavour compounds in MT-*Daqu*. Additionally, the extract was subjected to aroma extraction dilution analysis (AEDA) to identify key aroma-active compounds in MT-*Daqu*.

### Identification of volatile compounds

Volatile compounds in MT-*Daqu* were analyzed using a headspace solid-phase microextraction gas chromatography-mass spectrometry/olfactometry (HS–SPME–GC–MS/O) system, consisting of an Agilent 8890 GC coupled with a 7250 MS (Santa Clara, CA, USA) and an olfactometer (ODP C300, Gerstel, Germany). The GC–MS conditions followed the protocols established in previous experimental protocols (Deng et al. 2023). The shunt ratio between MS and ODP was 1:1, with the ODP transmission line and olfactory port temperatures set to 250 °C and 200 °C, respectively. GC-O analysis was conducted by three experienced evaluators, who had been trained to identify 40 volatile compounds at concentrations five times their odor thresholds in water prior to the experiment. Each MT-*Daqu* sample was analyzed in triplicate. Volatile compounds were categorized based on their mass spectra, referencing the NIST 20 mass spectral library database. Retention indices (RIs) were calculated using the retention times of n-alkanes (C7-C30) under the same GC–MS conditions. Final identification of the volatile compounds in MT-*Daqu* was achieved by correlating standard, odor, RI, and mass spectra data.

### Quantitative analysis

#### Semi-quantitative analysis

Semi-quantitative analysis of volatile compounds in MT-*Daqu* was conducted using HS–SPME–GC–MS, following a method described elsewhere (Yu et al. 2023). To perform this analysis, internal standards-volatile compounds that are absent from MT-*Daqu* but have known concentrations-were added to the samples. The concentrations of volatile compounds were calculated based on the peak areas of these internal standards in the GC–MS spectrum, without correcting for differences among the volatile compounds. In this study, n-butyl acetate and 2-acetylpyridine were chosen as internal standards for the quantification process.

#### Accurate quantitative analysis

LLE-GC–MS/MS was employed for the quantitative analysis of 42 aroma-active volatile compounds in MT-*Daqu*. Mixed standard solutions of these compounds were prepared according to the groupings described in Sect. "Sample collection". For Groups A and B, n-butyl acetate was used as the internal standard, while 2-acetylpyridine was used for Groups C and D. Each group of mixed standards was prepared at seven different concentration gradients. GC–MS analysis (7890 GC, 7100D Mass, Agilent Technologies, CA) was conducted that sampling was performed in shunt mode with a 10:1 ratio, the injection port temperature was set at 230 °C, the column temperature was programmed as follows: the initial temperature was programmed at 50 °C and held for 3 min; and then heated to150 °C at 3 °C /min and held for 5 min; finally heated to230 °C at 10 °C /min and held for 10 min; electron impact (EI) mass spectra were generated at 70 eV ionization energy, the MS source temperature was 150 °C. Multiple reaction monitoring (MRM) parameters used in this study are detailed in Table S2. Quantification of the volatile compounds in MT-*Daqu* was achieved using the internal standard method, with concentrations calculated from a quantitative curve generated using the linear least-squares method.

### Key aroma-active compounds analysis

#### Odor activity value (OAV)

The OAV of volatile compounds in MT-*Daqu* was calculated to assess the concentration of aroma-active compounds relative to their respective odor thresholds in water, following a method described elsewhere (Yu et al. 2024). The odor thresholds used in this study are based on values reported in a previous study (van Gemert 2011). OAV for aroma-active compounds in each sample group were computed according to the specified grouping requirements.

#### Aroma extraction dilution analysis (AEDA)

Based on the OAV, samples with the highest total OAV for aroma-active compounds within each group were selected for AEDA according to a method described in a previous study (Niu et al. 2020; Zhao et al. 2018). The volatile compound extracts from MT-*Daqu* were initially diluted fivefold and then further diluted in twofold steps for gas chromatography–olfactometry (GC-O) analysis under the previously described conditions. The maximum dilution factor represented the flavor dilution (FD) of each compound, with each dilution corresponding to its respective FD factor (Sha et al. 2016). Three replicates were performed for each sample, with a higher FD factor indicating a greater contribution of the compound to the overall aroma of the sample.

#### Aroma recombination experiments

Since wheat is the primary raw material used in MT-*Daqu* preparation, wheat flour was employed as a substrate for aroma activity recombination experiments (Yu et al. 2023). Based on the quantitative analysis of volatile compounds in MT-*Daqu* samples, aroma-active compounds with OAVs greater than 1, excluding those contributing to the raw material aroma, were selected and added to the wheat flour model to simulate sample aroma. Sensory evaluation and E-nose data analysis were conducted to compare the aroma profiles before and after recombination. Differences in the content of key aroma-active volatile compounds in each sample group informed the addition of specific aroma compounds, with sensory and E-nose analyses performed to assess changes in aroma profiles post-recombination.

### Statistical analysis

The content of volatile compounds and aroma scores in MT-*Daqu* samples were expressed as means ± standard deviations (SD). Differences among sample groups were assessed using Duncan’s ANOVA, conducted with SPSS v.14.0 (SPSS Inc., Chicago, IL, USA). Aroma score differences were visualized using GraphPad Prism 8 (GraphPad Software, Boston, USA). Principal component analysis (PCA), partial least squares discriminant analysis (PLS-DA), and visualizations such as complex heatmaps, radar maps, UpSet Venn diagrams, and Sankey diagrams were generated using RStudio software (https://posit.co/download/rstudio-server/).

## Results

### Sensory evaluation of MT-Daqu samples

**Fig. 1:**
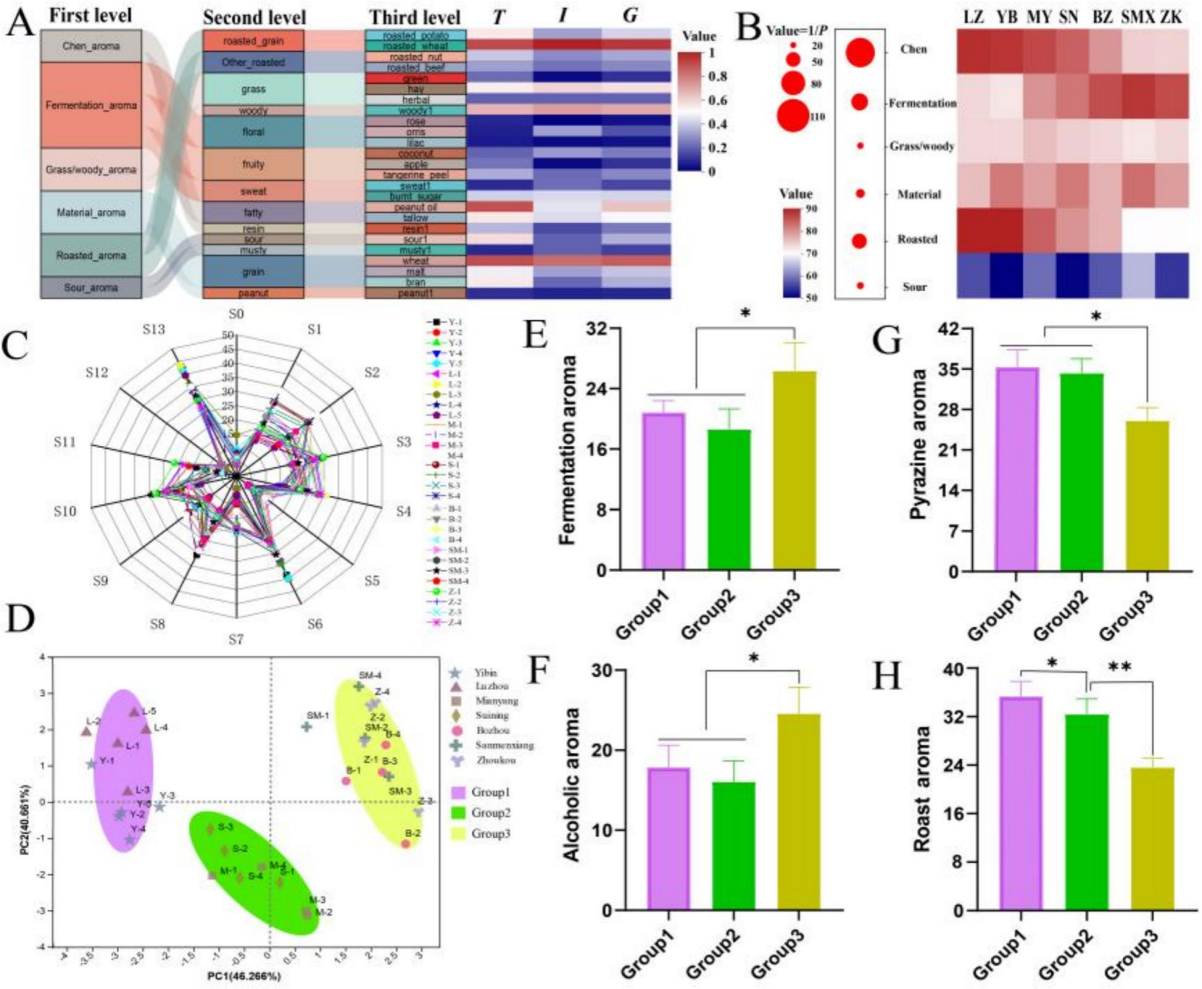
*Qu*-aroma evaluation of medium–high temperature *Daqu* (MT-*Daqu*) using sensory and Electronic nose (E-nose) analyses. **A** Sensory evaluation of *Qu*-aroma composition. **B** Aroma intensity analysis of MT-*Daqu* from different regions. **C** Radar map for the determination of the intensity of *Qu-*aroma using E-nose. **D** Principal component analysis (PCA) of the *Qu*-aroma intensity in different *Daqu* samples using E-nose. **E** Differences in fermentation aroma of MT-*Daqu* from different regions. **F** Differences in alcohol aroma of MT-*Daqu* from different regions. (**G**) Differences in pyrazine aroma of MT-*Daqu* from different regions. **H** Differences in roasted aroma of MT-*Daqu* from different regions.a “LZ”, “YB”, “MY”, “SN”, “BZ”, “SMX”, “ZK” indicate MT*-Daqu* samples from *Luzhou*, *Yibin*, *Mianyang*, *Suining*, *Bozhou*, *Sanmenxia*, and *Zhoukou* regions, respectively.b *T* indicates the ratio of times a descriptor is mentioned relative to the total possible mentions of that descriptor. c *I* is the sum of actual intensities assigned to a descriptor by the evaluation panel relative to the maximum possible intensity of that descriptor. d *G* is the geometric mean of the product of *T* and *I*

A sensory evaluation was conducted on 30 MT-*Daqu* samples from 7 SAB-producing regions. The *Qu*-aroma was categorized into three hierarchical levels. The first level included seven primary aromas: Chen aroma, fermentation aroma, grass/woody aroma, material aroma, roasted aroma, and sour aroma. The second and third levels included 13 and 25 additional aromas, respectively. The relationships among these hierarchical levels of *Qu*-aroma in MT-*Daqu* samples are illustrated in Fig. 1A. The roasted wheat aroma was the most frequently detected and intense across all MT-*Daqu* samples, followed by fatty and grain aromas. In contrast, rose and lilac aromas were observed with the lowest frequency and intensity. The sensory evaluation revealed that Chen aroma and roasted aroma were the predominant characteristics, with fermentation aroma serving as a secondary note.

A heatmap illustrating the sensory evaluation scores for MT-*Daqu* samples from various regions was created, with statistical differences assessed using Duncan’s ANOVA (Fig. 1B). With the exception of the sour aroma, which received an average score of approximately 60, all other *Qu*-aroma categories had scores exceeding 70. Significant regional differences were observed in the Chen aroma (*P* < *0.01*), as well as in the roasted aroma and fermentation aroma (*P* < *0.05*). Specifically, MT-*Daqu* samples from Sichuan displayed significantly higher scores for Chen and roasted aromas compared to samples from other regions (*P* < *0.05*). Conversely, Sichuan MT-*Daqu* samples had notably lower scores for fermentation aroma (*P* < *0.05*).

E-nose analysis utilizing 14 sensors was performed to further elucidate the *Qu*-aroma characteristics of MT-*Daqu* samples. A radar map, constructed from the aroma scores provided by the sensors, highlighted the overall aroma profile across the 30 samples, revealing notable similarities (Fig. 1C). Sensor S13, which detects pyrazines and lactone compounds, recorded the highest scores, followed by Sensor S6, which detects fatty and oil compounds. Conversely, Sensors S0, S5, S11, and S12, which detect ammonia, hydrogen, hydrogen sulfide, and sulfide, respectively, exhibited low scores. Principal Component Analysis (PCA) of the sensor data (Fig. 1D) showed that the first principal component (PC1) and the second principal component (PC2) accounted for 46.27% and 40.67% of the total variance, respectively. The PCA score plots revealed three distinct clusters of samples: Group 1, comprising MT-*Daqu* samples from Luzhou and Yibin; Group 2, consisting of samples from Mianyang and Suining; and Group 3, including samples from Bozhou, Sanmenxia, and Zhoukou. Statistical analysis indicated that Group 3 samples had significantly higher fermentation aroma (S1, S3, and S10 averaged) compared to the other groups (*P* < *0.05*). Similarly, the alcohol aroma (S3) followed this pattern (Figs. 1E and F).

In contrast, Groups 1 and 2 exhibited significantly higher levels of pyrazine aroma (S13) and roasted aroma (S4) compared to Group 3 (*P* < *0.05*). Group 1, in particular, demonstrated a significantly higher roasted aroma than Group 2 (*P* < *0.05*) (Fig. 1G and H). Overall, the combined sensory evaluation and E-nose analysis reveal that Sichuan MT-*Daqu* samples have lower fermentation aroma but higher roasted aroma compared to samples from other regions.

### Identification and semi-quantitative analysis of volatile compounds in MT-Daqu samples using HS-SPME-GC- MS/O

**Fig. 2:**
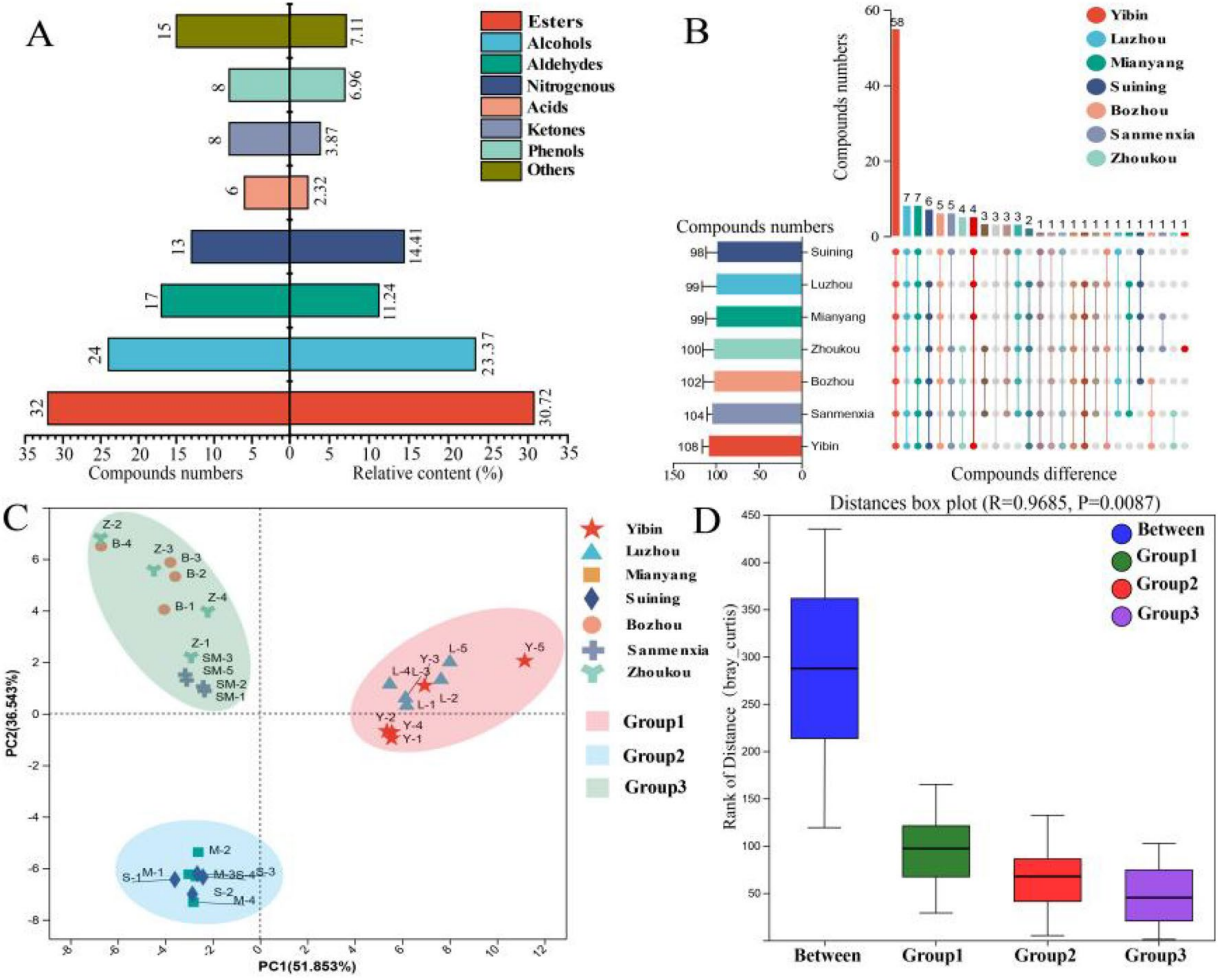
Identification and semi-quantitative analysis of volatile compounds in medium–high temperature *Daqu* (MT-*Daqu*)*.* **A** Volatile compounds and their relative content in MT-*Daqu*. **B** Differences in volatile compounds in MT-*Daqu* from different regions. (**C**) Principal component analysis (PCA) of volatile compounds in different *Daqu* samples. **D** Analysis of similarities (ANOSIM) of volatile compounds in different *Daqu* samples

Subsequently, the volatile compounds in MT-*Daqu* samples from seven SAB producing regions were analyzed using HS–SPME–GC–MS/O. A total of 123 volatile compounds were identified through a combined analysis of standards, odors, RI, and mass spectra results (Table S2). The identified volatile compounds were categorized as follows: 32 esters (30.72%), 24 alcohols (23.37%), 17 aldehydes (11.24%), 13 nitrogenous compounds (14.41%), 6 acids (2.32%), 8 ketones (3.87%), 8 phenols (6.96%), and 15 other components (7.11%) (Fig. 2A). An UpSet Venn diagram was constructed to visualize the similarities and differences in volatile compounds across different regions (Fig. 2B). There were no significant differences in the total number of volatile compounds among regions (*P* > *0.05*), with 58 volatile compounds being common to all MT-*Daqu* samples. Notably, limonene was detected exclusively in MT-*Daqu* samples from the Zhoukou region.

To further explore regional differences, Principal Component Analysis (PCA) based on semi-quantitative results of volatile compounds was performed. PC1 accounted for 51.85% and PC2 for 36.54% of the variance. PCA score plots indicated that MT-*Daqu* samples clustered into three distinct categories, which aligned with the grouping criteria observed in the E-nose analysis (Fig. 2C**).** To validate these groupings, ANOSIM analysis based on Bray–Curtis dissimilarity was conducted with 999 permutations (Fig. 2D). The results demonstrated significantly higher among-group differences compared to within-group differences (R = 0.9685, *P* < *0.01*), confirming the validity of the grouping criteria. Furthermore, GC-O analysis identified 42 aroma-active compounds among the 123 volatile compounds in MT-*Daqu* (Table 1). Of these, 32 were common across all samples. Although qualitative and semi-quantitative analyses indicated only minor differences in the number of identified volatile compounds across MT-*Daqu* samples from various regions, significant variations were observed in the content of specific volatile compounds, highlighting the need for further investigation into the absolute concentrations of *Qu*-aroma-active compounds.

**Table 1:** Key aroma-active compounds in medium–high temperature *Daqu* (MT-*Daqu*) samples from different producing regions

Aroma-active compounds	Order	Average content (mg/kg)			Odor threshold (mg/kg)	OAV					FD				
		Group1	Group2	Group3		Group1	Group2		Group3		Y-2	S-3		B-3	
Fermentation aroma (E-Nose:S1, S3and S10)															
Ethyl butyrate	Apple/pineapple	1.1114 ± 0.3830^a^	1.2590 ± 0.2876^a^	1.9758 ± 0.2564^b^	0.01^d^	111.14		125.9		197.58	8		8		32
1-Octanol	Tangerine peel	0.1236 ± 0.0567^a^	0.1450 ± 0.0391^a^	0.2419 ± 0.0520^b^	0.1^d^	1.24		1.5		2.42	0		0		2
γ-Nonanolactone	Coconut	0.5233 ± 0.1055^c^	0.3861 ± 0.1032^b^	0.2531 ± 0.0386^a^	0.1^d^	5.23		3.86		2.53	2		2		2
Ethyl isovalerate	Apple/pineapple	1.0694 ± 0.1388^a^	1.1960 ± 0.1813^a^	1.6602 ± 0.2174^b^	0.0011^d^	972.19		1087.27		1509.32	128		128		256
Ethyl valerate	Apple/pineapple	1.3887 ± 0.2270^a^	2.0396 ± 0.5080^b^	3.5533 ± 0.6492^c^	0.0087^d^	159.54		234.43		408.43	16		16		32
Ethyl caproate	Apple/pineapple	0.5097 ± 0.1227^a^	0.7718 ± 0.2726^a^	0.8611 ± 0.2726^a^	0.005^d^	101.95		154.37		172.21	16		16		64
Ethyl heptanoate	Pineapple	0.1254 ± 0.1009^a^	0.2719 ± 0.1330^b^	1.1038 ± 0.5091^c^	0.002^d^	62.69		135.94		551.91	16		32		128
Acetoin	Strawberry/milk	0.8502 ± 0.2811^a^	0.8164 ± 0.1393^a^	0.6947 ± 0.1466^a^	0.14^d^	6.07		5.83		3.53	2		2		0
Phenethyl acetate	Rose	0.2482 ± 0.1263^a^	0.6765 ± 0.0674^b^	1.4031 ± 0.3857^c^	0.25^d^	0		2.71		5.61	0		0		2
4-ethvlguaiacol	Lilac	1.4035 ± 0.2011^a^	1.2886 ± 0.2253^a^	1.0103 ± 0.3332^a^	0.122^d^	11.5		10.56		8.28	8		8		4
Benzyl alcohol	Sweet/floral	0.6008 ± 0.2474^a^	0.6187 ± 0.2901^a^	2.4387 ± 1.1337^b^	0.62^d^	0.96		0.98		3.93	0		0		2
Ethyl phenylacetate	Sweet/floral	0.8309 ± 0.2771^a^	1.3217 ± 0.2713^a^	1.2749 ± 0.4068^a^	0.15555^d^	5.34		8.5		8.2	2		2		4
Phenethyl alcohol	Honey	1.2812 ± 0.1437^b^	1.2856 ± 0.4124^b^	0.7939 ± 0.1979^a^	0.56^d^	2.29		2.3		1.42	2		2		2
1-Octen-3-ol	Mushrooms	0.1154 ± 0.0773^a^	0.1480 ± 0.0369^a^	0.2874 ± 0.0613^b^	0.03^d^	3.85		4.93		9.58	2		2		4
Chen aroma (E-Nose: S6)															
Butyraldehyde	Tallow	0.6733 ± 0.1722^a^	0.7148 ± 0.2545^a^	0.8759 ± 0.1701^a^	0.03^e^	22.44		23.82		29.2	16		16		8
Hexanal	Tallow	0.3383 ± 0.1003^b^	0.3009 ± 0.0485^b^	0.2066 ± 0.0773^a^	0.026^e^	13.03		11.59		7.96	32		32		16
1-Nonanal	Fatty	0.8527 ± 0.1008^c^	0.4749 ± 0.1420^b^	0.2062 ± 0.0578^a^	0.001^d^	852.67		474.86		206.05	512		256		128
Benzaldehyde	Peanut oil	0.1990 ± 0.0808^a^	0.2121 ± 0.0285^b^	0.1801 ± 0.0324^a^	0.188^d^	1.06		1.12		0.96	0		0		0
(*E*)-2-Nonenal	Resin	0.2288 ± 0.0994^a^	0.2349 ± 0.0363^a^	0.1694 ± 0.0533^a^	0.0004^d^	572		587.18		423.71	256		256		256
(*E*,*E*)-2,4-Decadienal	Fatty	0.1166 ± 0.0556^b^	0.1046 ± 0.0410^b^	0.0839 ± 0.0395^a^	0.00007^e^	1665.71		1495.14		1198.57	512		512		256
(*E*)-2-Octenal	Creamy	0.2161 ± 0.1239^a^	0.2151 ± 0.0677^a^	0.1949 ± 0.0731^a^	0.002^d^	108.04		107.54		97.46	32		32		32
Roasted aroma (E-Nose: S4 and S13)															
2,6-Dimethylpyrazine	Roasted potato	1.8292 ± 0.2775^c^	1.5057 ± 0.2711^b^	0.6408 ± 0.1685^a^	0.2^d^	9.14		7.53		3.2	8		4		2
3,5-Dimethyl-2-ethylpyrazine	Roasted wheat	0.5711 ± 0.1014^c^	0.4012 ± 0.0731^b^	0.2757 ± 0.0428^a^	0.001^e^	571.10		401.27		275.74	512		256		128
Tetramethylpyrazine	Roasted wheat	5.6993 ± 0.6897^c^	3.5067 ± 1.2154^b^	2.2465 ± 1.0072^a^	1^d^	5.7		3.51		2.25	4		2		2
4-Hydroxy-4-methoxystyrene	Roasted nut	0.2663 ± 0.1198^a^	0.3015 ± 0.1041^a^	0.2210 ± 0.0855^a^	0.012^d^	22.2		25.12		18.41	8		8		8
3-Methyl-1-butanol	burnt	0.6618 ± 0.1646^a^	1.0284 ± 0.3165^b^	2.8769 ± 0.4926^c^	0.2^d^	3.31		5.14		14.38	2		2		4
Sour aroma (E-Nose: S7)															
Butyric acid	Yoghurt	0.7224 ± 0.1655^a^	0.7508 ± 0.2084^a^	0.7343 ± 0.2992^a^	0.204^d^	3.54		3.68		3.6	2		2		2
3-Methylbutanoic acid	Sour	4.3275 ± 0.5806^a^	4.6666 ± 0.6751^a^	6.6713 ± 1.6755^b^	0.012^d^	360.06		380.89		550.59	128		128		256
Caproic acid	Sour	2.4061 ± 0.4655^a^	2.1496 ± 0.4794^a^	3.0092 ± 0.8521^a^	0.89^d^	1.58		2.42		5.63	0		0		4
Material aroma (E-Nose: S2)															
γ-Butyrolactone	Wheat	0.3003 ± 0.1417^a^	0.2520 ± 0.0580^a^	0.2925 ± 0.0771^a^	0.025^d^	12.01		10.08		11.7	8		8		8
Phenylethylene	Peanut	0.8339 ± 0.1067^a^	1.1450 ± 0.2866^a^	0.8155 ± 0.2082^a^	0.3^d^	2.78		3.82		2.72	2		2		2
Furfuryl alcohol	Bran	10.1176 ± 2.4482^a^	15.1995 ± 2.6097^b^	14.2387 ± 1.8447^b^	1^d^	10.12		15.2		14.24	2		4		4
Furfural	Bran	2.3400 ± 0.2487^b^	2.9294 ± 0.9712^b^	1.2068 ± 0.3522^a^	2^d^	1.17		1.46		0.6	2		2		0
3-Methylbutanal	Malt	3.9632 ± 0.4834^b^	4.3477 ± 0.6778^b^	3.0749 ± 0.6257^a^	0.0012^d^	3302.66		3623.11		2562.38	1024		1024		1024
Ethyl benzoate	Grain mildew	0.3799 ± 0.0953^a^	0.4549 ± 0.1537^b^	0.6894 ± 0.1685^b^	0.05556^d^	5.04		8.19		12.41	2		4		4
Grass/woody aroma (E-Nose: S8 and S9)															
1-Heptanol	Hay	0.1981 ± 0.0229^a^	0.1960 ± 0.0076^a^	0.5168 ± 0.0802^b^	0.0054^d^	36.69		36.31		95.71	32		32		64
2-Ethylhexanol	Hay	0.1606 ± 0.0971^a^	0.1917 ± 0.0620^a^	0.2408 ± 0.0989^a^	0.18^d^	0.88		1.06		1.34	0		0		2
Ethyl palmitate	Waxy	8.2212 ± 2.5742^a^	11.7717 ± 1.5440^b^	14.7565 ± 1.8499^c^	2^d^	4.41		5.39		7.38	2		2		4
Phenylacetaldehyde	Green	1.1306 ± 0.4116^a^	1.1492 ± 0.2973^a^	0.9830 ± 0.1099^a^	0.06^d^	21.66		19.15		16.38	16		16		8
2,3,5-Trimethylpyrazine	Earthy	0.7662 ± 0.1758^b^	0.6145 ± 0.1663^b^	0.3544 ± 0.1495^a^	0.35^d^	2.19		1.76		1.01	2		2		0
I-Caryophyllene	Woody	0.1255 ± 0.0466^a^	0.2549 ± 0.0337^a^	0.3479 ± 0.0944^b^	0.81^d^	0.31		1.7		2.32	0		0		2
Guaiacol	Woody	0.3564 ± 0.1307^b^	0.3940 ± 0.1344^b^	0.0747 ± 0.0206^a^	0.15^d^	2.38		2.63		0.5	2		2		0

1 The difference in the content of aroma active components in different groups is indicated by a, b and c, and different letters indicate significant difference (*P* < *0.05*); 2 d: Odor thresholds taken from a book (van Gemert 2011); 3 e: Odor thresholds taken from a paper(Wang et al. 2022); 4 Group 1 include MT-*Daqu* samples from *Luzhou* and *Yibin* regions; Group 2 include MT-*Daqu* samples from *Mianyang* and *Suining* regions; and Group 3 include MT-*Daqu* samples from *Bozhou*, *Sanmenxia*, and *Zhoukou* regions

### Quantitative analysis of volatile aroma-active compounds in MT-Daqu

**Fig. 3:**
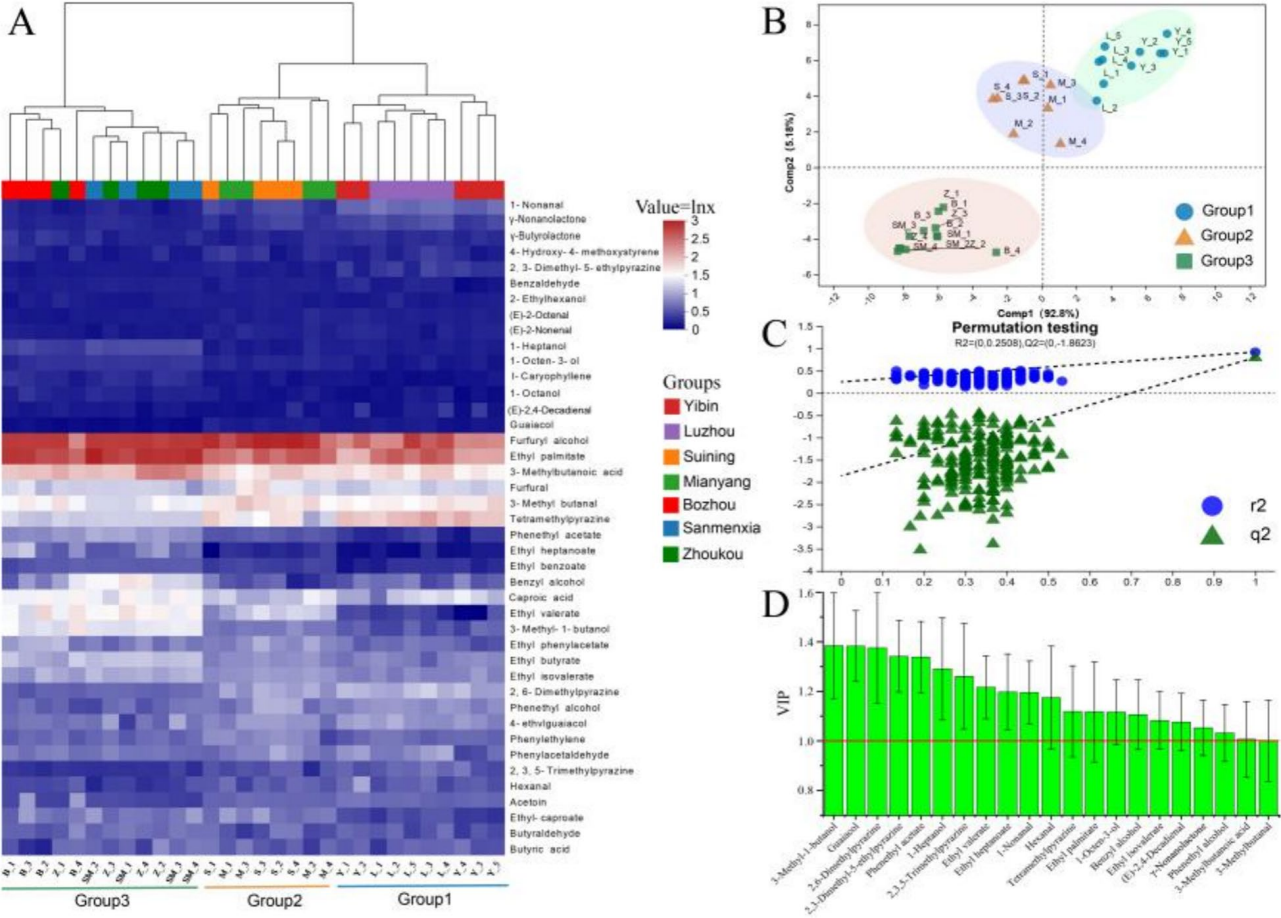
Analysis of the differences in aroma-active compound contents in medium–high temperature *Daqu* (MT-*Daqu*) samples from different producing regions. **A** Hierarchical clustering heatmap of aroma-active compound contents. **B** Partial Least Squares Discriminant Analysis (PLS-DA) plots of aroma-active compounds in MT-*Daqu* samples from different regions. **C** Performance of the permutation test. **D** Variable importance in projection (VIP) values of marker aroma-active compounds in MT-*Daqu* samples

LLE-GC–MS/MS was used to accurately quantify 42 aroma-active compounds in MT-*Daqu* samples. A cluster heatmap, based on the concentrations of these aroma-active compounds, is presented in Fig. 3A. Among the identified compounds, furfuryl alcohol exhibited the highest average concentration at 13.12 mg/kg, followed by ethyl palmitate (5.35 mg/kg), 3-methylbutanoic acid (3.73 mg/kg), tetramethylpyrazine (3.71 mg/kg), and 3-methylbutanal (4.35 mg/kg). Hierarchical clustering using Bray–Curtis dissimilarity revealed three distinct groups: Group 1, which included samples from Luzhou and Yibin; Group 2, which comprised samples from Suining and Mianyang; and Group 3, which consisted of samples from the remaining regions. This clustering pattern is consistent with previous analyses, and subsequent analyses focused on these three groups.

To further investigate the differences in aroma-active substances among the three groups, PLS-DA analysis was conducted. The results are shown in Fig. 3B. The PLS-DA model demonstrated an excellent fit, with R^2^X, R^2^Y, and Q^2^ values of 0.709, 0.989, and 0.956, respectively. The first component (Comp 1) accounted for 92.8% of the variance in the samples, while Comp 2 accounted for 5.18%. The PLS-DA score plots clearly separated the three MT-*Daqu* groups, although Groups 1 and 2 were found to be relatively close to each other. Permutation tests (n = 200) confirmed the quality of the model fit, with R^2^ = 0.2508 and Q^2^ = − 1.8623. The negative values for Q^2^ indicate that the model fit the data well (Fig. 3C).

Using Variable Importance in Projection (VIP) values greater than 1 to screen for key volatile compounds, 21 aroma-active compounds were identified as markers for discriminating the three sample groups (Fig. 3D). These included 5 esters (i.e., phenethyl acetate, ethyl valerate, ethyl heptanoate, ethyl palmitate, and ethyl isovalerate), 5 alcohols (i.e., 3-methyl-1-butanol, 1-heptanol, 1-octen-3-ol, benzyl alcohol, and phenethyl alcohol), 5 aldehydes (i.e., 1-nonanal, γ-nonanolactone, 3-methylbutanal, hexanal, and (*E*)-2,4-decadienal), 4 nitrogenous components (i.e., 2,6-dimethylpyrazine, 2,3,5-trimethylpyrazine, tetramethylpyrazine, and 2,3-dimethyl-5-ethylpyrazine), and 2 other compounds (i.e., 3-methylbutanoic acid and guaiacol). In addition, there were 21 components that did not show significant differences among the three sample groups, including 3 esters, 3 alcohols, 3 aldehydes, 2 nitrogenous components, 2 acids, and 8 other compounds. The quantitative analysis highlighted significant differences in the levels of these aroma-active compounds between MT-*Daqu* samples from Sichuan and those from other regions, as well as among samples from various SAB-producing areas within Sichuan.

### Analysis of key aroma-active compounds in MT-Daqu* from different regions*

Based on the results of sensory evaluation, E-nose analysis, and aroma-active compounds analyses, the 42 identified aroma-active compounds in MT-*Daqu* were classified into several aroma categories: fermentation aroma (14 compounds), Chen aroma (7 compounds), roasted aroma (5 compounds), sour aroma (3 compounds), material aroma (6 compounds), and grass/woody aroma (7 compounds). MT-*Daqu* samples were categorized into three groups, as previously determined in Sect. "Quantitative analysis of volatile aroma-active compounds in MT-Daqu". The average content of aroma-active compounds within each group was calculated, and differences between groups were assessed using ANOVA.

Additionally, OAVs of aroma-active compounds were calculated based on their odor thresholds in water. One representative sample from each group was selected for AEDA to determine the FD factors for the 42 aroma-active compounds (Table 1). Significant differences in the content of 10 aroma-active compounds were observed among the three groups (*P* < *0.05*), with 12 aroma-active compounds showing significant differences between Sichuan province samples and those from other regions (*P* < *0.05*). Notably, except for furfuryl alcohol and furfural, these differences were found in aroma-active compounds with VIP > 1. The OAVs of 3-methylbutanal, which contributes to malt aroma, were highest in Group 3, reaching 3302.66. Among fermentation aromas, ethyl isovalerate had the highest OAVs, reaching 1509.32 in Group 3. Other compounds such as ethyl valerate, ethyl heptanoate, and ethyl caproate also showed higher OAVs in Group 3 compared to Groups 1 and 2.

In contrast, the OAVs of compounds such as 1-nonanal, (E)-2-nonenal, and (E)-2,4-decadienal, which contribute to Chen aroma, were higher in Group 1 and Group 2 compared to Group 3. Additionally, the OAVs of 2,3-dimethyl-5-ethylpyrazine, which contributes to roasted aroma, were highest in Group 1, followed by Group 2 and Group 3. The FD factors of the 42 aroma-active compounds for Y-2, S-3, and B-3 samples, as determined by the AEDA experiment, were similar to those of OAVs in MT-*Daqu* samples from different regions.

**Fig. 4:**
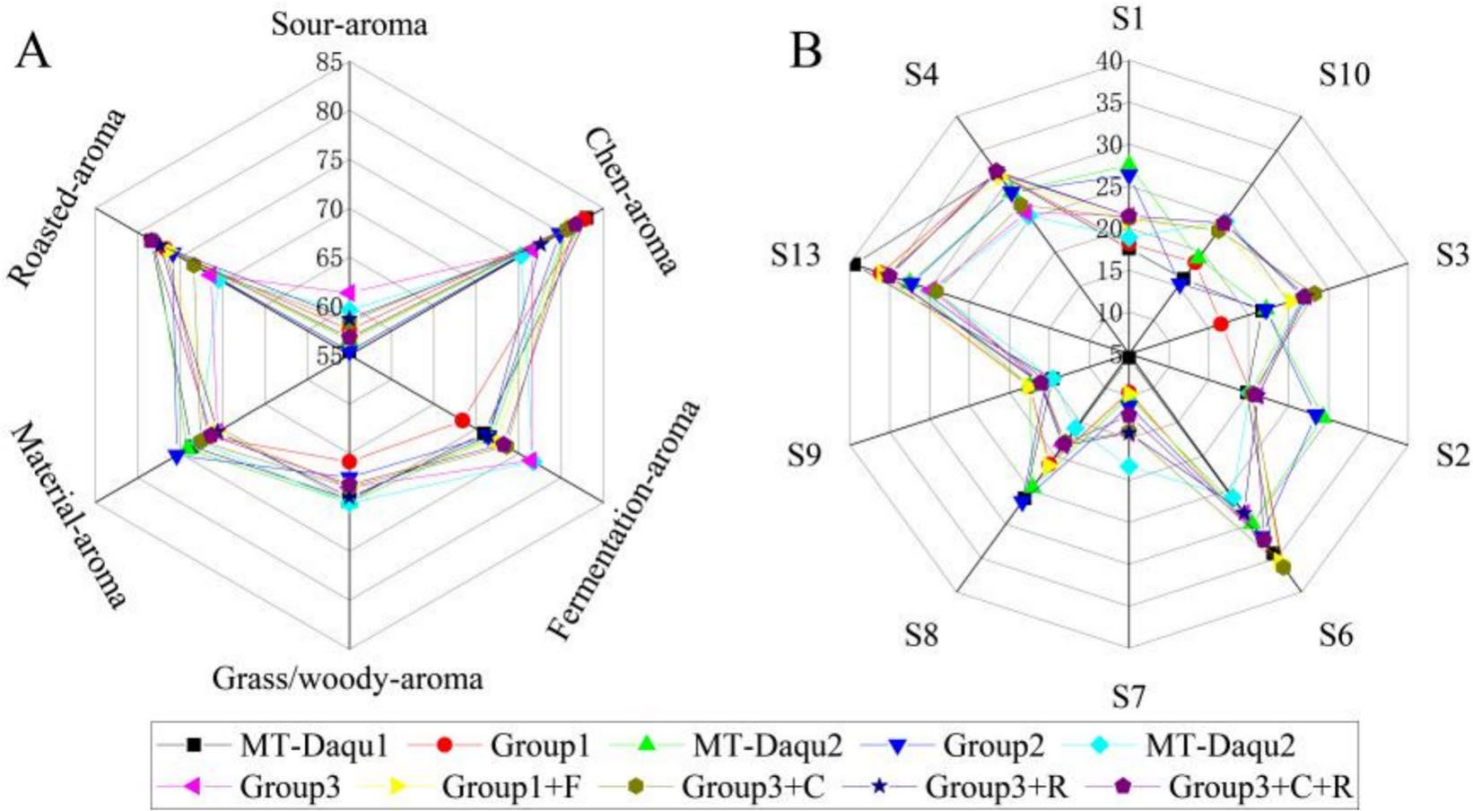
*Qu*-aroma recombination experiments for medium–high temperature *Daqu* (MT-*Daqu*) from different producing regions. **A** Sensory evaluation radar chart. **B** Electronic nose odor identification radar map

Based on the clustering pattern of samples and the content of aroma-active compounds, recombinant experiments (Group 1, Group 2, and Group 3) were recombinant experiments were performed using samples from Group 1, Group 2, and Group 3 to create combined models (MT-*Daqu* 1, MT-*Daqu* 2, and MT-*Daqu* 3). The average scores for these recombination models were analyzed through sensory evaluation (Fig. 4A) and E-nose analysis (Fig. 4B). The results revealed that the aroma profiles of the recombinant models closely matched those of the original samples, demonstrating that the key aroma-active compounds in MT-*Daqu* samples were accurately characterized.

Based on the differences observed in the content of aroma-active compounds, OAVs, and FD factors in MT-*Daqu* samples, three groups of components were selected for recombination experiments: 9 fermentation-aroma compounds, 4 Chen-aroma compounds, and 4 roasted-aroma compounds (Table 2). The addition of these three groups of aroma-active compounds improved the scores for their respective aroma categories in the recombinant models (Fig. 4A and B), confirming that these compounds are key contributors to the aroma profile of MT-*Daqu*. Notably, Chen aroma and roasted aroma were more pronounced in Group 1 samples compared to Group 3, whereas fermentation aroma compounds were more prominent in Group 3.

**Table 2:** Content of aroma-active compounds added in recombination experiments

Aroma-active compounds	Content (mg/Kg)	Aroma-active compounds	Content (mg/Kg)
Fermentation aroma (Group1 + F)		Chen aroma (Group3 + C)	
Ethyl butyrate	0.8644	Hexanal	0.3094
Ethyl isovalerate	0.5908	1-Nonanal	0.6465
Ethyl valerate	2.1646	(*E*)-2-Nonenal	0.0594
Ethyl caproate	0.3514	(*E*,*E*)-2,4-decadienal	0.0347
Ethyl heptanoate	0.9784	Roasted aroma (Group3 + R)	
Phenethyl acetate	1.1549	2,6-Dimethylpyrazine	1.1884
Benzyl alcohol	1.8379	2,3-Dimethyl-5-ethylpyrazine	0.2954
1-Octen-3-ol	0.172	Tetramethylpyrazine	3.4528
Ethyl phenylacetate	0.444	4-Hydroxy-4-methoxystyrene	0.0453

Group 1 + F, adding partial fermentation aroma compounds to Group 1; Group 3 + C, adding partial Chen aroma compounds to Group3; Group 3 + R, adding partial roasted aroma compounds to Grou

Further experiments involved adding partial fermentation aroma compounds to Group 1 (Group 1 + F), adding partial Chen aroma to Group 3 (Group 3 + C), and adding partial roasted aroma to Group 3 (Group 3 + R). The specific added contents are detailed in Table 2. The aroma components and their contents were significantly different in Group 1 and Group 3. Aroma evaluation after recombination revealed that the fermentation aroma score for the Group 1 + F model was lower than that of Group 3. Additionally, the fermentation aroma scores for Group 3 + C, Group 3 + R, and Group 3 + C + R decreased, suggesting that Chen aroma and roasted aroma compounds may mask the fermentation aroma in MT-*Daqu*.

## Discussion

In the present study, 30 MT-*Daqu* samples from 7 *SAB*-producing regions in China were analyzed, identifying 7 primary aromas contributing to the *Qu*-aroma of MT-*Daqu*: Chen aroma, fermentation aroma, grass/woody aroma, material aroma, roasted aroma, and sour aroma. These primary aromas were further subdivided into 25 specific sub-aromas. While these findings are consistent with existing literature, there are some differences in classification compared to previous studies (S.-B. Yang et al. 2024a, b). Notably, other aromas such as unpleasant, rancid/cheesy, and fermented odors have been reported in past research. However, the sensory evaluators in this study found these aromas were not consistently present across all MT-*Daqu* samples and therefore did not consider them characteristic of *Qu*-aroma, despite their occasional detection.

In this study, a total of 123 volatile compounds were identified in MT-*Daqu* samples from various regions, with 42 of these compounds found to be aroma-active. This finding contrasts with previous reports that identified 111 and 90 volatile compounds in MT-*Daqu* (Wang et al. 2022; S.-B. Yang et al. 2024a, b). The higher number of identified compounds in the present study can be attributed to more extensive sample coverage and representativeness. Moreover, the use of improved sample pretreatment methods and advanced detection equipment likely contributed to a more thorough analysis of the *Qu*-aroma compounds in MT-*Daqu* samples, such as GC × GC-TOF/MS, UPLC-Q-TOF/MS, GC-IMS and other devices(Miao et al. 2024; Yang et al. 2021).

From an aroma-active perspective, the fermentation aroma category was the most diverse, encompassing 14 compounds primarily including alcohols and esters. However, sensory evaluation scores for fermentation aroma were lower compared to those for Chen aroma and roasted aroma categories. This discrepancy can be attributed to two main factors: (i) several fermentation aroma-active compounds, such as 1-octanol, γ-nonanolactone, and benzyl alcohol, have low OAVs and FD factors; and (ii) the potential masking effect of Chen aroma and roasted aroma on fermentation aroma. This masking effect has been documented in previous studies, which explored the factors influencing aroma release of ester alcohols in *Baijiu* (Niu et al. 2018; Qin et al. 2024; S. Yang et al. 2024a, b).

Chen aroma is a significant component of MT-*Daqu*, primarily composed of fatty and oil aldehydes. Key aroma-active compounds associated with Chen aroma include hexanal, 1-nonanal, (*E*)-2-nonenal, (*E*,*E*)-2,4-decadienal, and butyraldehyde, which aligns with previous research on Chen aroma compounds in MT-*Daqu* (S.-B. Yang et al. 2024a, b). Roasted aroma comprises five aroma-active compounds, including three pyrazines, 4-hydroxy-4-methoxystyrene, and 3-methyl-1-butanol. Pyrazines are crucial to roasted aroma (Deng et al. 2023; S.-B. Yang et al. 2024a, b), formed during the high-temperature fermentation (55–60 °C) of wheat flour over extended periods (8–15 days), which facilitate Maillard reactions. Moreover, microorganisms such as *Bacillus* contribute to the synthesis of pyrazines (Liu et al. 2023a, b, c).

The sour aroma, primarily attributed to 3-methylbutanoic acid, is carefully controlled in MT-*Daqu* production. High-quality MT-*Daqu* is expected to have minimal sour aroma, which is reflected in the low sour taste scores in sensory evaluations. Material aroma, which includes compounds such as 3-methylbutanal, γ-butyrolactone, and furfuryl alcohol, likely originates from the wheat or auxiliary materials used in MT-*Daqu* preparation (S.-B. Yang et al. 2024a, b). Grass/woody aroma, characterized by compounds like 1-heptanol, 2-ethylhexanol, phenylacetaldehyde, 2,3,5-trimethylpyrazine (grass aroma), I-caryophyllene, and guaiacol (wood aroma), has lower OAVs and FD factors, resulting in lower sensory evaluation scores. These aromas may also originate from the auxiliary tools and instruments used during MT-*Daqu* production.

This study provides a detailed analysis of the *Qu*-aroma of MT-*Daqu* by examining the composition and characteristics of aroma-active compounds. The findings offer valuable insights into the distinct features of the *Qu*-aroma and lay the groundwork for further research on how it impacts the overall aroma of *SAB*. The study also assessed regional differences in *Qu*-aroma characteristics and aroma-active compounds, highlighting significant variations across different production areas. For instance, MT-*Daqu* samples from Sichuan were found to have higher scores for Chen aroma and roasted aroma compared to samples from other regions, whereas the fermentation aroma showed the opposite trend. Additionally, six major aroma-active compounds-ethyl isovalerate, ethyl heptanoate, hexanal, 1-nonanal, (*E*,*E*)-2,4-decadienal, and 2,3-dimethyl-5-ethylpyrazine-were consistently identified across all MT-*Daqu* samples. Despite significant regional differences in the content of certain aroma compounds (*P* < *0.05*), their overall contribution to the aroma was not markedly different. For example, compounds like 3-methyl-1-butanol, γ-Nonanolactone, Phenethyl alcohol, ethyl palmitate, and tetramethylpyrazine, while varying in content, did not significantly alter the overall aroma profile, potentially due to their high aroma thresholds (Yu et al. 2024).

The observed differences in aroma-active compounds among MT-*Daqu* samples from various regions effectively account for the variations in *Qu*-aroma characteristics. These regional differences in *Qu*-aroma result from a combination of factors, including environmental conditions and production parameters. While some of the aroma in MT-*Daqu* originates directly from raw materials and tools-such as material aroma and grass/woody aroma-the majority of aroma compounds undergo transformations through chemical reactions and microbial interactions. Previous research has demonstrated that the microbial community structure of MT-*Daqu* varies by region, with environmental conditions being a key driver of microbial evolution (Ma et al. 2022a, b; Ma et al. 2022a, b). These microbial differences largely contribute to the variations in volatile compounds found in MT-*Daqu*. For example, yeast has been shown to significantly influence the production of alcohols and esters (Li et al. 2024), while *Bacillus* is crucial for synthesizing nitrogen compounds (Liu et al. 2023a, b, c). Additionally, environmental conditions impact the chemical reactions in MT-*Daqu*. Changes in temperature and humidity, for instance, can affect Maillard reaction and fatty acid oxidation processes (Cao et al. 2024; Hu et al. 2024), thereby influencing the formation of Chen aroma and roasted aroma compounds in MT-*Daqu*.

To better understand the regional differences in the *Qu*-aroma of MT-*Daqu* from various producing areas, future research should focus on the production conditions and mechanisms influencing *Qu*-aroma development. Investigating these factors will provide valuable insights for optimizing production processes with a flavor-oriented approach, ultimately enhancing the quality of MT-*Daqu*.

## Conclusions

In this study, the aroma composition and key aroma-active compounds of MT-*Daqu* from seven different production areas were thoroughly analyzed, revealing significant regional variations in the aroma profiles of MT-*Daqu*. The *Qu*-aroma of MT-*Daqu* samples was characterized using six broad categories, including fermentation aroma, Chen aroma, roasted aroma, sour aroma, material aroma and grass/woody aroma, and twenty-five specific aroma descriptors. Chen aroma and roasted aroma were notably more prevalent in MT-*Daqu* samples from Sichuan compared to those from other regions (*P* < *0.05*), and the law of fermented aroma was opposite. A total of 123 volatile compounds were identified, 42 of which were recognized as aroma-active compounds. Among these, 21 aroma-active compounds were identified as markers distinguishing Sichuan MT-*Daqu* from those produced in non-Sichuan regions, showing significant differences in their concentrations (*P* < *0.05*), included 5 esters, 5 alcohols, 5 aldehydes, 4 nitrogenous components, and 2 other compounds. Six major aroma-active compounds were found to significantly influence the aroma differences among MT-*Daqu* samples. This study underscores the regional variations in the *Qu*-aroma of MT-*Daqu* and offers insights that could guide flavor-oriented optimization of the production process to enhance MT-*Daqu*’s quality.

## Supplementary Information

Supplementary Material 1.

## Data Availability

Not applicable.
