# Supplementary material for: Characterization of Qu-aroma of medium–high temperature Daqu from different production areas using sensory evaluation, E-nose, and GC–MS/O analysis
Source: Bioresour Bioprocess. 2025 Apr 26;12(1):40. doi: 10.1186/s40643-025-00863-y (PMC12033147; doi:10.1186/s40643-025-00863-y)
Supplement: Supplementary file 1 — Supplementary Material 1. [file 40643_2025_863_MOESM1_ESM.docx]

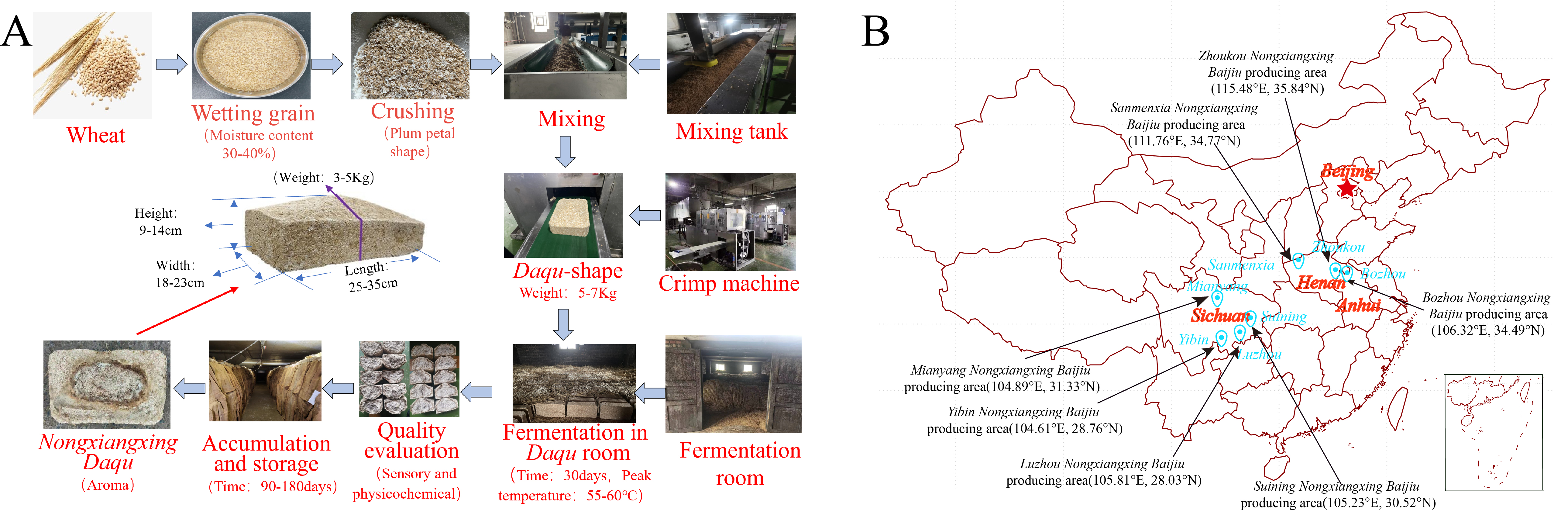


**Figure S1 Production process of MT-*Daqu*（A）and the sampling coordinates of the MT-*Daqu* from different *SAB*-producing region(B)**

Note：1、MT-*Daqu* is made from wheat, and brick-shaped blocks with a length of 25-35cm, a width of 18-28cm, a height of 9-14cm, and a weight of 3-5 kg. 2、Sichuan is the largest SAB-producing region, South Sichuan production region (*Luzhou* and *Yibin*, the former State Administration of Quality Supervision, Inspection and Quarantine approved the "Golden Triangle of Chinese liquor");Northeast Sichuan production region (*Suining* and *Mianyang*), followed by Anhui (B*ozhou*) and Henan ( *Sanmenxia* and *Zhoukou*) production region.

**Table S1 The sensors of E-nose system**

| NO. | Odor attributes |
| --- | --- |
| S0 | Ammonia, amines |
| S1 | Aromatic compounds |
| S2 | Lactone, chaff |
| S3 | Alcohol, organic solvents |
| S4 | Volatile compounds of food cooking |
| S5 | Hydrogen |
| S6 | Aldehydes, fats and oil |
| S7 | Sour, organic solvents |
| S8 | Alkanes, flammable gas |
| S9 | Sterol, triterpenes, smell of hay |
| S10 | Volatile compounds of food fermentation |
| S11 | Sulphide |
| S12 | Hydrogen sulfur |
| S13 | Pyrazine, Nitrogen-containing volatile compounds |

Note：The E-nose system has a total of 24 array sensors, of which 10 sensors are external complements. The system can be used for the detection of fermented food, baked food and meat, and has been analyzed many samples, such as *baijiu*, tea and tea leaves, baked bread and roast meat.

**Table S2 The volatile components of MT- *Daqu***

| NO.^a^ | Volatile components | CAS | Molecular formula | Molecular weight | RI^b^ | Identification method^c^ | Aroma^d^ | selected ions (m/z)^e^ |
| --- | --- | --- | --- | --- | --- | --- | --- | --- |
| 1 | Dimethylamine | 124-40-3 | C_2_H_7_N | 45.1 | 721 | MS,RI |  |  |
| 2 | Trimethyl amine | 75-50-3 | C_3_H_9_N | 59.1 | 776 | MS,RI |  |  |
| 3 | Acrolein | 107-02-8 | C_3_H_4_O | 56.1 | 821 | MS,RI |  |  |
| 4 | 2-Methylpropanal | 78-84-2 | C4H8O | 72.1 | 836 | MS,RI |  |  |
| 5 | Acetone | 67-64-1 | C_3_H_6_O | 58.1 | 851 | S,MS,RI |  |  |
| 6 | Ethyl acetate | 141-78-6 | C_4_H_8_O_2_ | 88.1 | 896 | S,MS,RI |  |  |
| 7 | Acetal | 105-57-7 | C_6_H1_4_O_2_ | 118.2 | 906 | MS,RI |  |  |
| 8 | Methanol | 67-56-1 | CH_4_O | 32.2 | 913 | S,MS,RI |  |  |
| 9 | 1,2-Propanediol | 4254-15-3 | C_3_H_8_O_2_ | 76.1 | 936 | S,MS,RI |  |  |
| 10 | Ethyl butyrate | 105-54-4 | C_6_H_12_O_2_ | 116.2 | 1040 | S,MS,RI,O | fruit | 60,71,88,116 |
| 11 | Ethyl isovalerate | 108-64-5 | C_7_H_14_O_2_ | 130.2 | 1064 | MS,RI,O | fruit | 59,70,88,130 |
| 12 | Butyraldehyde | 123-72-8 | C_4_H_8_O | 72.1 | 1074 | S,MS,RI,O | pungent | 27,44,72 |
| 13 | Hexanal | 66-25-1 | C_6_H_12_O | 100.1 | 1086 | S,MS,RI,O | grassy | 43,56,72,82 |
| 14 | Isobutanol | 78-83-1 | C_4_H_10_O | 74.1 | 1099 | S,MS,RI |  |  |
| 15 | 3-Methylbutyraldehyde | 590-86-3 | C_5_H_10_O | 86.1 | 1116 | S,MS,RI,O | wheat | 44,58,71,86 |
| 16 | Isoamyl acetate | 123-92-2 | C_7_H1_4_O_2_ | 130.2 | 1121 | MS,RI |  |  |
| 17 | Ethyl valerate | 539-82-2 | C_7_H_14_O_2_ | 130.2 | 1131 | S,MS,RI,O | fruit | 57,73,88,103 |
| 18 | Limonene | 138-86-3 | C_10_H_16_ | 136.2 | 1181 | MS,RI |  |  |
| 19 | Dodecane | 112-40-3 | C_12_H_26_ | 170 | 1193 | MS,RI |  |  |
| 20 | 3-Methyl-1-butanol | 123-51-3 | C_5_H_12_O | 88.1 | 1202 | S,MS,RI,O | alcohol | 42,55,70 |
| 21 | 2-Ethylbutyl methacrylate | 5138-86-3 | C10H18O2 | 170.2 | 1209 | MS,RI |  |  |
| 22 | 2-Pentylfuran | 3777-69-3 | C_9_H_14_O | 138.2 | 1229 | S,MS,RI |  |  |
| 23 | Ethyl caproate | 123-66-0 | C_8_H_16_O_2_ | 144.2 | 1235 | S,MS,RI,O | fruit | 60,73,88,99 |
| 24 | 1-Pentanol | 71-41-0 | C_5_H_12_O | 88.2 | 1248 | S,MS,RI, |  |  |
| 25 | Phenylethylene | 100-42-5 | C_8_H_8_ | 104.1 | 1257 | S,MS,RI,O | ripe wheat | 39,78,104 |
| 26 | 3-Octanone | 106-68-3 | C_8_H_16_O | 128.2 | 1264 | MS,RI | butter | 57,72,99,128 |
| 27 | 2-Methylpyrazine | 109-08-0 | C_5_H_6_N_2_ | 94.1 | 1270 | S,MS,RI |  |  |
| 28 | Hexyl acetate | 142-92-7 | C_8_H_16_O_2_ | 144.2 | 1277 | MS,RI |  |  |
| 29 | Acetoin | 513-86-0 | C_4_H_8_O_2_ | 88.1 | 1286 | S,MS,RI,O | Strawberries | 45,55,73,88 |
| 30 | 2-Octanone | 111-13-7 | C_8_H_16_O | 128.2 | 1295 | MS,RI |  |  |
| 31 | 3-Methyl-3-buten-1-ol | 556-82-1 | C_5_H_10_O | 86.1 | 1311 | S,MS,RI |  |  |
| 32 | 2,5-Dimethyl pyrazine | 123-32-0 | C_6_H_8_N_2_ | 108.1 | 1323 | S,MS,RI |  |  |
| 33 | 2,6-Dimethylpyrazine | 108-50-9 | C_6_H_8_N_2_ | 108.1 | 1330 | S,MS,RI,O | Baked potato | 42,67,81,108 |
| 34 | Ethyl heptanoate | 106-30-9 | C_9_H_18_O_2_ | 158.2 | 1337 | S,MS,RI，O | fruit | 77,117,148 |
| 35 | 6-Methylhept-5-en-2-one | 110-93-0 | C_8_H_14_O | 126.2 | 1344 | MS,RI |  |  |
| 36 | 2,3-Dimethylpyrazine | 5910-89-4 | C_6_H_8_N_2_ | 108.1 | 1374 | S,MS,RI |  |  |
| 37 | Ethyl lactate | 97-64-3 | C_5_H_10_O_3_ | 118.1 | 1352 | S,MS,RI |  |  |
| 38 | Hexyl formate | 629-33-4 | C_7_H_14_O_2_ | 130.2 | 1358 | MS,RI |  |  |
| 39 | 1-Hexanol | 111-27-3 | C_6_H_14_O | 102.2 | 1365 | S,MS,RI |  |  |
| 40 | 3-Octanol | 589-98-0 | C8H18O | 130.2 | 1375 | MS,RI |  |  |
| 41 | 1-Nonanal | 124-19-6 | C_9_H_18_O | 142.2 | 1386 | S,MS,RI,O | Fatty/oily | 41,57,70,98,142 |
| 42 | 2-Nonanone | 821-55-6 | C_9_H_18_O | 142.2 | 1390 | MS,RI |  |  |
| 43 | 2-ethyl-6-methylPyrazine | 13925-03-6 | C_7_H_10_N_2_ | 122.1 | 1395 | MS,RI,O |  |  |
| 44 | 2,3,5-Trimethylpyrazine | 14667-55-1 | C_7_H_10_N_2_ | 122.2 | 1405 | S,MS,RI,O | earthy | 42,54,81,122 |
| 45 | Butyl caproate | 626-82-4 | C_10_H_20_O_2_ | 172.2 | 1425 | MS,RI |  |  |
| 46 | Ethyl caprylate | 106-32-1 | C_10_H_20_O_2_ | 172.2 | 1434 | S,MS,RI |  |  |
| 47 | (E)-2-Octenal | 2548-87-0 | C_8_H_14_O | 126.2 | 1439 | S,MS,RI，O | grassy/fatty | 41,55,83,97,126 |
| 48 | 1-Octen-3-ol | 3391-86-4 | C_8_H_16_O | 128.2 | 1446 | S,MS,RI,O | floral | 43,57,72,85,99 |
| 49 | 3-Furaldehyde | 498-60-2 | C_5_H_4_O_2_ | 96.1 | 1453 | S,MS,RI | bitter almond |  |
| 50 | 1-Heptanol | 111-70-6 | C_7_H_16_O | 116.2 | 1457 | S,MS,RI，O | hay | 43,56,70,98,116 |
| 51 | Acetic acid | 64-19-7 | C_2_H_4_O_2_ | 60.1 | 1461 | S,MS,RI |  |  |
| 52 | 2,3-Dimethyl-5-ethylpyrazine | 15707-34-3 | C_8_H_12_N_2_ | 136.1 | 1469 | MS,RI |  |  |
| 53 | 3,5-Dimethyl-2-ethylpyrazine | 13925-07-0 | C_8_H_12_N_2_ | 136.1 | 1476 | S,MS,RI,O | roasted | 42,54,80,108,136 |
| 54 | Tetramethylpyrazine | 1124-11-4 | C_8_H_12_N_2_ | 136.2 | 1484 | S,MS,RI,O | roasted | 42,54,81,95,136 |
| 55 | 2-Ethenyl-6-methylpyrazine | 13925-09-2 | C_7_H_8_N_2_ | 119.5 | 1491 | MS,RI |  |  |
| 56 | 2-Ethylhexanol | 104-76-7 | C_8_H_18_O | 130.2 | 1499 | S,MS,RI |  |  |
| 57 | Decanal | 112-31-2 | C_10_H_20_O | 156.2 | 1506 | S,MS,RI |  |  |
| 58 | 2,3,5-Trimethyl-6-ethylpyrazine | 17398-16-2 | C_9_H_14_N_2_ | 150.2 | 1517 | MS,RI |  |  |
| 59 | Benzaldehyde | 100-52-7 | C_7_H_6_O | 106.1 | 1528 | S,MS,RI,O | bitter almond | 45,77,106 |
| 60 | （E)-2-Nonenal | 18829-56-6 | C_9_H_16_O | 140.2 | 1535 | S,MS,RI,O | grassy/fatty | 43,55,70,83,111 |
| 61 | Ethyl nonanoate | 123-29-5 | C_11_H_22_O_2_ | 186.3 | 1542 | MS,RI |  |  |
| 62 | Linalool | 78-70-6 | C_10_H_18_O | 154.2 | 1558 | MS,RI |  |  |
| 63 | 1-Octanol | 111-87-5 | C_8_H_18_O | 130.2 | 1565 | S,MS,RI |  |  |
| 64 | 2,3-Butanediol | 513-85-9 | C_4_H_10_O_2_ | 90.1 | 1573 | S,MS,RI,O |  |  |
| 65 | Ethyl 3-methylthiopropionate | 13327-56-5 | C_6_H_12_O_2_S | 148.2 | 1580 | MS,RI |  |  |
| 66 | 5-Methyl furfural | 620-02-0 | C_6_H_6_O_2_ | 110.1 | 1589 | MS,RI |  |  |
| 67 | I-Caryophyllene | 87-44-5 | C_15_H_24_ | 204.3 | 1598 | MS,RI,O | woody | 69,93,105,133,189 |
| 68 | Hexyl hexanoate | 6378-65-0 | C_12_H_24_O_2_ | 200.3 | 1606 | MS,RI |  |  |
| 69 | Trans-2-Octen-1-ol | 18409-17-1 | C_8_H_16_O | 128.2 | 1619 | MS,RI |  |  |
| 70 | Butyric acid | 107-92-6 | C_4_H_8_O_2_ | 88.1 | 1625 | S,MS,RI,O | Sour | 29,41,60,73,88 |
| 71 | Ethyl caprate | 110-38-3 | C_12_H_24_O_2_ | 200.3 | 1631 | MS,RI |  |  |
| 72 | 6-Methyl-1-octanol | 110453-78-6 | C_9_H_20_O | 144.2 | 1637 | MS,RI |  |  |
| 73 | Phenylacetaldehyde | 122-78-1 | C_8_H_8_O | 120.1 | 1643 | S,MS,RI,O | penicillin/grassy | 65,91,120 |
| 74 | γ-Butyrolactone | 96-48-0 | C_4_H_6_O_2_ | 86.1 | 1646 | MS,RI,O | wheat | 28,42,56,86 |
| 75 | Acetophenone | 98-86-2 | C_8_H_8_O | 120.1 | 1654 | S,MS,RI |  |  |
| 76 | Ethyl benzoate | 93-89-0 | C_9_H_10_O_2_ | 150.1 | 1661 | S,MS,RI,O | musty smell | 51,77,105,122 |
| 77 | Furfuryl alcohol | 98-00-0 | C_5_H_6_O_2_ | 98.1 | 1665 | S,MS,RI，O | burnt/caramel | 41,53,69,81,98 |
| 78 | Diethyl succinate | 123-25-1 | C_8_H_14_O_4_ | 174.2 | 1673 | MS,RI |  |  |
| 79 | α-Caryophyllene | 6753-98-6 | C_15_H_24_ | 204.3 | 1680 | MS,RI |  |  |
| 80 | 3-Methylbutanoic acid | 503-74-2 | C_5_H_10_O_2_ | 102.1 | 1689 | S,MS,RI，O | acidity | 43,60,87,102 |
| 81 | Heptadecane | 629-78-7 | C_17_H_36_ | 240.5 | 1699 | S,MS,RI |  |  |
| 82 | α-Terpineol | 98-55-5 | C_10_H_18_O | 154.2 | 1708 | MS,RI,O |  |  |
| 83 | 3-Methylthiopropanol | 505-10-2 | C_4_H_10_OS | 106.2 | 1715 | MS,RI |  |  |
| 84 | 3-methyl-2(5H)-Furanone | 22122-36-7 | C_5_H_6_O_2_ | 98.1 | 1728 | MS,RI |  |  |
| 85 | 1,4-Dimethoxybenzene | 150-78-7 | C_8_H_10_O_2_ | 138.1 | 1739 | S,MS,RI |  |  |
| 86 | Naphthalene | 91-20-3 | C_10_H_8_ | 128.1 | 1748 | S,MS,RI |  |  |
| 87 | 1,3-Dimethoxybenzene | 151-10-0 | C_8_H_10_O_2_ | 138.1 | 1753 | S,MS,RI |  |  |
| 88 | α-Cumyl alcohol | 617-94-7 | C_9_H_12_O | 136.2 | 1765 | MS,RI |  |  |
| 89 | Methyl salicylate | 119-36-8 | C_8_H_8_O_3_ | 152.2 | 1776 | MS,RI |  |  |
| 90 | Ethyl phenylacetate | 101-97-3 | C_10_H_12_O_2_ | 164.2 | 1794 | S,MS,RI,O | sweat | 39,51,65,91,119 |
| 91 | Butyrophenone | 495-40-9 | C_10_H_12_O | 148.2 | 1802 | S,MS,RI |  |  |
| 92 | (E)-2,4-Decadienal | 25152-84-5 | C_10_H_16_O | 152.2 | 1811 | S,MS,RI，O | fatty/grassy | 41,67,81,95,123,152 |
| 93 | Ethyl salicylate | 118-61-6 | C_9_H_10_O_3_ | 166.2 | 1820 | MS,RI |  |  |
| 94 | Phenethyl acetate | 103-45-7 | C_10_H_12_O_2_ | 164.2 | 1826 | S,MS,RI，O | roses | 43,78,91,104 |
| 95 | Ethyl laurate | 106-33-2 | C_14_H_28_O_2_ | 228.3 | 1839 | S,MS,RI |  |  |
| 96 | Caproic acid | 142-62-1 | C_6_H_12_O_2_ | 116.1 | 1856 | S,MS,RI,O | sour | 41,60,73,87 |
| 97 | 3-phenylfuran | 13679-41-9 | C_10_H_8_O | 144.1 | 1869 | MS,RI |  |  |
| 98 | Guaiacol | 90-05-1 | C_7_H_8_O_2_ | 124.1 | 1875 | S,MS,RI,O | woody | 39,53,81,109,124 |
| 99 | Benzyl alcohol | 100-51-6 | C_7_H_8_O | 108.1 | 1887 | S,MS,RI,O | floral | 51,79,91,108 |
| 100 | Phenethyl alcohol | 60-12-8 | C_8_H_10_O | 122.1 | 1914 | S,MS,RI,O | honey | 51,65,77,91,103,122 |
| 101 | 2-Phenyl-2-butenal | 4411-89-6 | C_10_H_10_O | 146.1 | 1926 | S,MS,RI，O |  |  |
| 102 | Heptanoic acid | 111-14-8 | C_7_H_14_O_2_ | 130.1 | 1942 | S,MS,RI，O |  |  |
| 103 | Benzothiazole | 95-16-9 | C_7_H_5_NS | 135.1 | 1956 | MS,RI |  |  |
| 104 | 2-Methoxy-4-methylphenol | 93-51-6 | C_8_H_10_O_2_ | 138.2 | 1962 | MS,RI |  |  |
| 105 | 2-Acetylpyrrole | 1072-83-9 | C_6_H_7_NO | 109.1 | 1973 | MS,RI |  |  |
| 106 | Phenethyl isovalerate | 140-26-1 | C_13_H_18_O_2_ | 206.3 | 1984 | MS,RI |  |  |
| 107 | Phenol | 108-95-2 | C_6_H_6_O | 94.1 | 1993 | S,MS,RI |  |  |
| 108 | 4-ethvlguaiacol | 2785-89-9 | C_9_H_12_O_2_ | 152.1 | 2009 | S,MS,RI,O | clove | 51,65,91,109,152 |
| 109 | γ-Nonanolactone | 104-61-0 | C_9_H_16_O_2_ | 156.2 | 2017 | MS,RI,O | coconut | 55,71,85,99,138 |
| 110 | Ethyl myristate | 124-06-1 | C_16_H_32_O_2_ | 256.4 | 2032 | S,MS,RI |  |  |
| 111 | Ethyl cinnamate | 103-36-6 | C_11_H_12_O_2_ | 176.2 | 2052 | MS,RI |  |  |
| 112 | Octanoic acid | 124-07-2 | C_8_H_16_O_2_ | 144.2 | 2077 | S,MS,RI |  |  |
| 113 | p-Cresol | 106-44-5 | C_7_H_8_O | 108.1 | 2093 | MS,RI |  |  |
| 114 | Cocal | 21834-92-4 | C13H16O | 188.3 | 2106 | MS,RI |  |  |
| 115 | 4-Ethylphenol | 123-07-9 | C_8_H_10_O | 122.1 | 2168 | S,MS,RI |  |  |
| 116 | 4-Hydroxy-3-methoxystyrene | 7786-61-0 | C_9_H_10_O_2_ | 150.1 | 2184 | S,MS,RI,O | bake | 51,77,89,107,150 |
| 117 | Ethyl palmitate | 628-97-7 | C_18_H_36_O_2_ | 284.4 | 2260 | S,MS,RI,O | wax | 61,73,88,101,157 |
| 118 | Ethyl hexadecenoate | 54546-22-4 | C_18_H_34_O_2_ | 282.4 | 2283 | S,MS,RI |  |  |
| 119 | Dibenzofuran | 132-64-9 | C_12_H_8_O | 168.2 | 2298 | MS,RI |  |  |
| 120 | 2,4-Di-t-butylphenol | 96-76-4 | C_14_H_22_O | 206.3 | 2321 | MS,RI |  |  |
| 121 | Ethyl octadecanoate | 111-61-5 | C_20_H_40_O_2_ | 312.5 | 2411 | MS,RI |  |  |
| 122 | Ethyl oleate | 111-62-6 | C_20_H_38_O_2_ | 310.5 | 2465 | S,MS,RI |  |  |
| 123 | Octadecanoic acid,ethyl ester | 111-61-5 | C_20_H_40_O_2_ | 312.5 | 2484 | S,MS,RI |  |  |

Note：1、a:Volatile components were numbered consecutively according to RI values (DB-WAX); 2、b：Retention indices；3、c：MS, RI, O, and S were represented for identifying by mass spectra (MS), retention indices (RI), odor (O), and standard compounds (S), respectively.4、d：Odor perception of each aroma-active compound that was detected at the sniffing port；5、e：The largest molecular weight was the mother ion, and the rest of ions was for quantitative.
